# Immune microenvironment and immunotherapy for chordoma

**DOI:** 10.3389/fonc.2024.1374249

**Published:** 2024-06-24

**Authors:** Yujia Chen, Hongwei Zhang

**Affiliations:** Department of Neurosurgery, Sanbo Brain Hospital, Capital Medical University, Beijing, China

**Keywords:** chordoma, immune microenvironment, immunotherapy, immune checkpoint, clinical trial

## Abstract

Chordoma, as a rare, low-grade malignant tumor that tends to occur in the midline of the body, grows slowly but often severely invades surrounding tissues and bones. Due to the severe invasion and damage to the surrounding tissues, chordoma is difficult to be gross totally resected in surgery, and the progression of the residual tumor is often unavoidable. Besides, the tumor is insensitive to conventional radiotherapy and chemotherapy, thus finding effective treatment methods for chordoma is urgent. Nowadays, immunotherapy has made a series of breakthroughs and shown good therapeutic effects in kinds of tumors, which brings new insights into tumors without effective treatment strategies. With the deepening of research on immunotherapy, some studies focused on the immune microenvironment of chordoma have been published, most of them concentrated on the infiltration of immune cells, the expression of tumor-specific antigen or the immune checkpoint expression. On this basis, a series of immunotherapy studies of chordoma are under way, some of which have shown encouraging results. In this review, we reviewed the research about immune microenvironment and immunotherapy for chordoma, combined with the existing clinical trials data, hoping to clarify the frontiers and limitations of chordoma immune research, and provide reference for follow-up immunotherapy research on chordoma.

## 1 Introduction

Chordoma is a relatively rare, low-grade malignant tumor originating from residual notochord tissue during embryonic development, accounting for 1% to 4% of malignant bone tumors, with an incidence of about 0.08/100,000 people in the population (1–3). It is prone to occur in the midline structures of the human body, with common sites including the skull base (35%), sacrococcygeal region (50%), and the spine (15%) (2). Occasionally, it can be found in metastatic lesions such as ribs, lungs, brain, or spinal cord (4–6).

Although chordoma has a slow proliferation rate, it often causes severe destruction of surrounding soft tissue and bone during tumor progression, which makes it extremely difficult to completely resected and the 5-year progression-free survival (PFS) rate is only 59.2% (3, 7). Besides, chordoma is not sensitive to conventional radiotherapy and chemotherapy, which requires higher radiation doses (usually greater than 74 Gy) to achieve therapeutic goals, meanwhile also increasing the risk of radiational damage for the surrounding tissues (8, 9). Multiple phase II clinical trials have confirmed that targeted therapy against growth factors could significantly benefit patients overall and also significantly prolong their survival. However, further phase III clinical studies are still lacking to support the clinical application of these targeted treatment strategies (10–12). Such limited treatment options often lead chordoma patients to undergo multiple cycles of “surgery-recurrence” and face a dilemma of “incurable” at last when surgery is no longer possible (13). It’s an urgent need to explore new treatments for chordoma.

Previous studies found that Brachyury is a tumor-specific antigen in chordoma, leading to some studies designed tumor vaccines targeting this protein and conducted clinical trials accordingly, which became the beginning of research on immunotherapy of chordoma (14, 15). Since then, more research on the characteristics of the immune microenvironment in chordoma has continued to emerge, which has also provided a theoretical basis for more clinical immunotherapy, especially the immune checkpoint inhibitor (ICI) therapy (16).

In this review, we reviewed the existing evidence on chordoma immune research, clarified the state of the immune microenvironment in chordoma, presented the frontier and focus issues of chordoma immunotherapy clinical research hoping to provide a guideline for follow-up immunotherapy research on chordoma.

## 2 Immune microenvironment characteristics of chordoma

**Figure 1:**
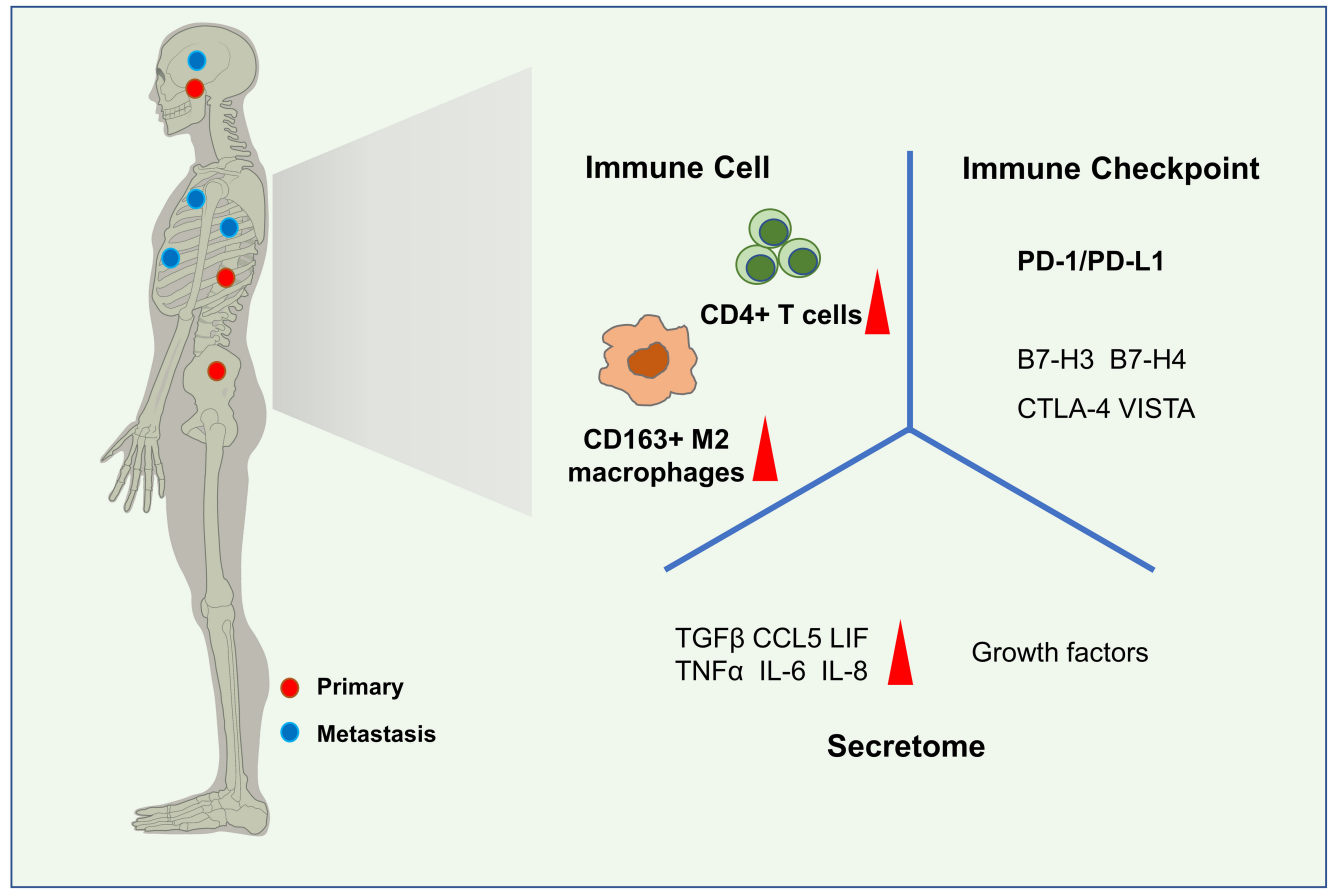
Immune microenvironment characteristics of chordoma. Common sites of chordoma include the skull base, sacrum, and spine. Metastatic lesions may be found in the ribs, lungs, brain, or spinal cord(left). In the immune microenvironment of chordoma, CD4+ T cells and CD163+ M2 macrophages are the most abundant immune cells subsets. Kinds of secretome,such as TGFβ, TNFα, CCL5, IL-8, IL-6, LIF are highly expressed in chordomas and some of them play important roles, as well as growth factors. The expression of some immune checkpoints,especially PD-1/PD-L1 has been ddetected in the tumor tissue and peripheral blood.

Due to the low incidence, research on the immune microenvironment of chordoma had been relatively lacking compared to other bone tumors. While the basic research technology developing and the emphasis on tumor immune characteristics increasing, the research on the immune microenvironment of chordoma, especially the infiltration of immune cells, the secretion of cytokines and chemokines in tumor microenvironment (TME), and the expression of immune checkpoints in chordoma had made significant progress nowadays (**Figure 1**).

### 2.1 Immune cell in chordoma

#### 2.1.1 The infiltration of immune cell

The infiltration of immune cells are an important part of the immune microenvironment and the cellular basis for immunotherapy. Several studies illustrated the characteristic of immune cells infiltration in chordoma detected by different methods. Shalin performed immunohistochemical (IHC) staining of 24 chordoma specimens and found CD4+ T lymphocyte infiltration in 21 patients and CD8+ T lymphocyte infiltration in only 11 patients (17). Dridi also found that macrophages and CD4+ T cells were the most abundant immune cell groups in chordoma tissues, followed by CD8+ T cells (18). Several studies further analyzed the subgroups of the infiltrated CD4+ T cells and macrophages in chordoma. Wanru conducted single-cell sequencing on 6 chordoma specimens and found that chordoma tissue had abundant regulatory T cells and M2 macrophages (19). Morimoto et al.’s study of 54 skull base chordomas also showed that there were a large number of CD163+ M2 macrophages and Foxp3+ regulatory T cells in skull base chordomas (20). These studies indicated that there was a large infiltration of CD4+ T cells and macrophages in chordoma, especially regulatory T cells and M2 macrophages.

Although previous studies had been consistent on the degree of infiltration of macrophages, there was inconsistent on the extent of tumor-infiltrated lymphocytes (TIL) in chordoma in the literature. Dancsok detected and calculated the number of infiltrating immune cells per unit area in 28 chordoma cases, they found that macrophages were the highest density infiltrating cell population, with CD163+ M2 macrophages far more than CD86+ M1 macrophages, but both of them were much more than TILs, which differed from the presence of large CD4+ T cell infiltrated in chordomas mentioned in other studies (21). This inconsistence might be due to different experimental assay methods or materials, which need further research to elucidate.

#### 2.1.2 The distribution of immune cell

Previous research also focused on the spatial distribution of immune cells in chordoma. Diana et al. used multiple immunofluorescence (mIF) to observe the distribution of immune cells in 75 chordoma patients. They distinguished the immune cells distribution between tumor parenchyma and the extracellular matrix, observed that macrophage infiltration density was significantly higher in the tumor parenchyma than in the matrix, while helper T cells, cytotoxic T cells and regulatory T cells were more abundant in the tumor matrix than in the tumor. NK cells in chordoma were rare and hardly activated (22). Additionally, since these chordoma tissues came from patients in different locations and stages, the study also found that there was no difference in the distribution of immune cells in chordomas among different locations and stages (22). This research suggested that immune cell infiltration in chordoma was relatively stable, and myeloid cells were more closely distributed to tumor cells than T cells (22).

#### 2.1.3 The correlation between immune cell and prognosis

Several studies had analyzed the correlation between specific immune cell subsets and prognosis in patients with chordoma. Dridi found that the infiltration of CD8+ T cell was a protective factor for chordoma (18). Mingxiang et al. also found that there was a significant positive correlation between CD3+ T lymphocyte infiltration and the degree of tumor invasion, overall survival (OS) and local relapse-free survival (LRFS) in 54 spinal chordomas, and the density of CD4+ T lymphocytes was significantly associated with OS and LRFS, while the density of Foxp3+ regulatory T cells and CD8+ T cells was associated with OS (23, 24). Jinpeng et al.’s study on TIL staining in 93 chordomas found that the content of TIM3+ TILs was associated with the degree of chordoma invasion and was an independent prognostic risk factor for LRFS and OS (25). Furthermore, studies demonstrated that the patient’s tumor/stromal ratio and CD8+/Foxp3+ ratio were independent risk factors for prognosis (26, 27).

However, previous studies had mainly observed the correlation between the infiltration of TILs and the prognosis of patients in chordoma, but few of them studied on the correlation between the infiltration of myeloid cells and the prognosis of patients. Only Morimoto’s study of 54 skull base chordomas showed that the infiltration of CD163+ M2 macrophages and Foxp3+ regulatory T cells were significantly higher in rapid-progressing chordomas than in slow-progressing chordomas, suggesting that M2 macrophages and regulatory T cells might have a potential role in the progression of chordomas (20).

### 2.2 Secretome in chordoma

Studies of chordoma immune cells had confirmed the presence of a large number of immune cells infiltrated in chordomas, indicating the presence of complex cell-to-cell interactions. In addition to the direct contact between cells, many cell interactions also require the mediation of cytokines, chemokines, growth factors, and even metabolic products, collectively known as secretome. Although previous studies had not reported on the expression and secretion of growth factors in chordoma, several phase II clinical studies had confirmed that inhibiting growth factors could prolong the survival of patients with chordoma, demonstrating the crucial role of growth factors in chordoma (10–12).

Recently, it had been discovered that kinds of cytokines and chemokines, such as TGFβ, TNFα, CCL5, IL-8, IL-6 and LIF, also as important mediators of cellular interactions, had been found to be highly expressed in chordomas and play important roles in the immune microenvironment.

#### 2.2.1 The TGFβ family in chordoma

Studies on TGFβ in chordoma were relatively abundant, suggesting that the TGFβ pathway played a crucial role in chordoma. Studies showed that the loss of heterozygosity for 1p36 presented in 75%-90% of chordoma samples, which suppressed the expression of RUNX (an inhibitor gene of TGFβ) and in turn led to the activation of the TGFβ pathway in chordoma cells (28–31). Stefan et al. performed RNA-seq on 5 chordoma samples and 3 chordoma cell lines, compared them with notochord tissue and found that TGFβ was highly expressed in chordoma and that inhibition of the TGFβ pathway could attenuate chordoma cell growth (32). Another study performed single-cell sequencing analysis on 6 chordoma cases and revealed that the TGF-β signaling pathway was not only essential on the progression of chordoma, but also induced the malignant progression of tumor cells to extracellular matrix (ECM), maintained the tumor stem cells self-renewal, promoted the change of extracellular matrix (EMT) and the transformation of CD4+T cells to Treg+ cells in chordoma (19). A more detailed study of TGFβ found that the expression level of TGFB1 was an important factor affecting the prognosis of skull base chordoma (33), and the down-regulation of TGFB3—a gene that acted opposite to TGFB1—might be a key factor for chordoma tumorigenesis (34). As for the source of TGFβ, single-cell sequencing studies had shown that TGFβ in chordoma was mainly secreted by fibroblasts and macrophages (19). Overall, the above results suggested that inhibition of TGFβ secretion or activation might be a potential target for chordoma treatment.

#### 2.2.2 The CCL5 in chordoma

The first research on CCL5 in chordoma were just published, demonstrating a critical role for CCL5 in tumor-macrophage interactions in chordoma. Jiuhui et al. constructed a tumor-macrophage co-culture model to simulate the *in vivo* situation of chordoma, and then screened the expression levels of RNA and protein in MUG-chor1 chordoma cells after co-culture with macrophages. The expression level of CCL5 was significantly increased in chordoma cells, and experiments confirmed that CCL5 could induce the M2 polarization of macrophages and promote the invasion and migration of chordoma cells. It was also confirmed that the expression of CCL5 in recurrent chordoma was significantly higher than that in the original tumor (35). Although there was only this one research on CCL5 in chordoma, the study clearly confirmed the secretory source of CCL5 and verified its role in the chordoma microenvironment.

#### 2.2.3 Other cytokines and chemokines in chordoma

Previous studies suggested that some cytokines and chemokines, including TNFα, LIF, IL-6 and IL-8 played important roles in the immune microenvironment of chordoma, but relatively few studies had been conducted.

Sukru et al. stained some kinds of cytokines in 14 chordoma tissues and found that the expression level of TNFα in chordoma was negatively correlated with the survival time of patients, and positively correlated with higher LIF and PD-L1expression in chordoma (36). And LIF, as a member of the IL-6 cytokine family, was inversely correlated with poorer overall survival and was shown to inhibit tumor inflammatory responses (37). However, the source of their secretion in chordoma and how they played a role in influencing patient outcomes remained unclear.

As for IL-6 and IL-8, studies have shown that the expression levels of IL-6 and IL-8 in the serum of patients with chordoma are significantly higher than those of normal people (38), and their secretion levels in chordoma cells are significantly increased after co-culture with macrophages *in vitro (* 35), indicating that their secretion was affected by tumor-immune cell interactions, but the mechanism was still unclear. Based on the finding, a Phase I clinical trial of IL-8 monoclonal antibody for chordoma treatment had been carried out and showed good safety, but the efficacy had yet to be verified (39).

Overall, although we had some understanding of cytokines and chemokines expression, cellular interactions in the immune microenvironment of chordoma, there was still a lack of in-depth research on the source of cytokine secretion, the specific mechanisms by which cytokines mediated cellular interactions, and the expression levels in chordoma. Further addressing these issues was crucial for seeking new potentially effective treatments for chordoma.

### 2.3 Immune checkpoint in chordoma

#### 2.3.1 PD-1/PD-L1 in chordoma

Due to the excellent therapeutic efficacy of ICI therapy in a wide range of tumors, the expression of immune checkpoints was one of the hotspots in current immune research in chordoma. Among them, PD-1/PD-L1 was the focus of immune checkpoint research in chordoma.

Previous studies on the PD-1/PD-L1 in chordoma mainly focused on the expression levels and sites, but the number of published studies was not substantial. Mathios detected the expression of PD-1 and PD-1 ligands in 10 chordoma specimens and 3 chordoma cell lines by reverse transcription-polymerase chain reaction (RT-PCR) and found that PD-1 expressed on TILs, while PD-L1 expressed on both TILs and tumor-associated macrophages (TAMs) in 3/10 tumor specimens (40). Laura detected two pediatric poorly differentiated chordomas using whole-genome, transcriptome and whole-genome bisulfite sequencing (WGBS) and multiplex immunohistochemistry, revealed the expression of PD-L1in both patient samples (41). Dride stained 81 chordoma specimens and found that PD-L1 was detected on inflammatory cells but not expressed on tumor cells in 26% of patients (18). Interestingly, while other studies have found that PD-L1 is only expressed on immune cells, Chao et al.’s immunofluorescence staining study on 92 chordomas showed that PD-L1 expression could also be found in patient tumor cells, which was inconsistent to other studies, and needed further studies to explain the difference.

Besides, studies had found that the expression of PD-L1 correlated to the prognosis of patients in chordoma. The degree of PD-L1+ immune cells correlated to tumor blood vessel density and lower PD-L1 expression correlated to the better patient prognosis (18, 42). Both PD-1+ cell density and PD-L1+ cell density were significant risk factors for local PFS and OS in chordoma (23, 26, 43).

#### 2.3.2 Other immune checkpoints in chordoma

In addition to the PD-1/PD-L1, research on the others immune checkpoints in chordoma were relatively scarce. He et al.’s study on thirty-two spinal chordomas showed that the expression level of CTLA-4 in chordoma tissues was higher than that in nucleus pulposus tissue, and it was a risk factor for OS (44). Long et al. detected the expression of six kind of cell surface receptors, including PD-L1, B7 -H3, B7-H4 and VISTA which could be targeted by CAR-T in forty-five skull base chordoma samples, the results showed that B7-H3 had the highest positive rate (7/45, 16%) in samples, and the positive rate was ranked as B7-H3 > PD-L1 > VISTA = B7-H4, suggesting that B7-H3 was a good target for CAR-T treatment of chordoma (45). The study also designed a CAR-T targeting B7-H3 and verified its good effectiveness against chordoma *in vitro* experiments.

#### 2.3.3 Soluble immune checkpoints in chordoma

Some studies had attempted to detect the expression of soluble immune checkpoints in peripheral blood of chordoma patients. Kushlinskii et al. conducted ELISA detection for soluble PD-1 (sPD-1), soluble PD-L1 (sPD-L1) and soluble VISTA (sVISTA) in the peripheral blood of chordoma patients and found that there were higher expression of sPD-L1 and lower expression of sVISTA in chordoma patient than in control, no matter the tumor location, stage, tumor type, patient age and gender (46, 47), suggesting that chordoma could affect the expression of soluble immune checkpoints and might affect the immune status of the whole body.

Overall, studies on immune checkpoints in chordoma had showed that there certainly were kinds of immune checkpoints expressed in chordoma tissues. Although the expression level was general low, the existing preliminary studies still suggested that there were effective targets for ICIs therapy or other immunotherapies targeting these immune checkpoints (such as CAR-T therapy) in chordoma, and related treatments may be effective against chordoma.

## 3 Research on immunotherapy of chordoma

The tumor immune microenvironment is closely related to tumor immunotherapy. Deeply understanding the immune microenvironment is essential to develop and optimize immunotherapy strategies and find more effective treatment methods. As the understanding of the immune microenvironment of chordoma deepens, research on immunotherapy for chordoma also continues to grow.

**Figure 2:**
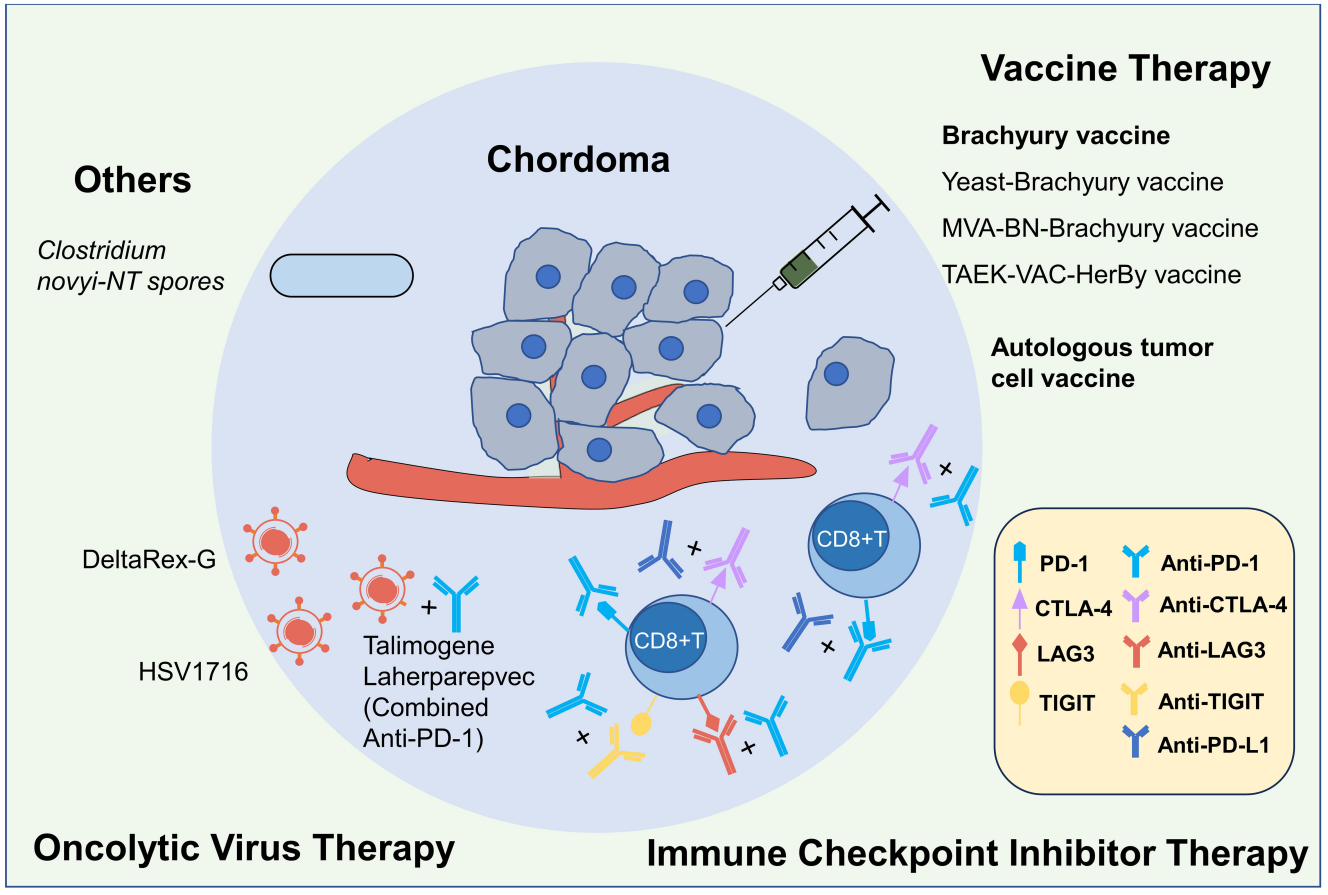
Immunotherapies for chordoma. Currently, the immunotherapy research on chordoma mainly includes four categories: vaccine, immune checkpoint inhibitor (ICI), oncolytic virus and others, and previous published studies primarily focused on the first two categories. In terms of the Brachyury vaccine research, the safety of the yeast-derived Brachyury vaccine and the MVA-BN-Brachyury-TRICOM vaccine has been identified, but their efficacy still requires further research. The phase I clinical trial of TAEK-VAC-HerBy vaccine was ongoing (NCT04246671). There have been one case report on autologous tumor cell vaccine. Previous ICIs studies mostly been based on individual cases, lacking large-scale studies, and its afty and efficacy varies. Currently, there are multiple clinical studies underway exploring immune checkpoint inhibitors with different ICI regimens and combination therapies. Research on oncolytic virus and other treatments is relatively limited, but several relevant clinical trials are also underway.

Currently, kinds of clinical trials on immunotherapy of chordoma were ongoing, and the published results mainly involved in vaccine therapy and ICI therapy, while other immunotherapy methods such as CAR-T therapy, oncolytic virus therapy, had not been reported in the literature so far. Due to the paucity of published literature, we searched for information on clinical trials in chordoma. As of July 3, 2023, there were 66 registered chordoma clinical trials on the Clinical trial website (https://www.clinicaltrials.gov) and sixteen of them were effective and involved immunotherapy, which was far more than published immune studies, including 9 trials of ICI therapy, 3 trials of vaccine therapy, 2 trials of oncolytic virus therapy and 2 trials of other immunotherapies (**Figure 2**, **Table 1**).

**Table 1:** Immunotherapy Clinical Trials in Chordoma.

Classification	NCT Number	Study Title	Interventions	Age	Phase	Enrollment	Combination with Other Therapy
Immune Checkpoint Inhibitor Therapy	NCT04416568	Study of Nivolumab and Ipilimumab in Children and Young Adults with INI1-Negative Cancers	Nivolumab (Anti-PD-1 antibody), Ipilimumab (Anti-CTLA-4 antibody)	Child, Adult	Phase2	45	/
	NCT03623854	Nivolumab and Relatlimab in Treating Participants with Advanced Chordoma	Nivolumab (Anti-PD-1 antibody), Relatlimab (Anti-LAG-3 antibody)	Child, Adult, Older adult	Phase2	10	/
	NCT03058289	A Phase 1/2 Safety Study of Intratumorally Dosed INT230-6	Anti-PD-1 antibody, Anti-CTLA-4 antibody, INT230-6 (A novel drug)	Adult, Older adult	Phase1, Phase2	110	Combination with a novel, experimental drug, INT230-6.
	NCT02936102	A Study of FAZ053 Single Agent and in Combination with PDR001 in Patients with Advanced Malignancies.	FAZ053 (Anti-PD-L1 antibody), PDR001 (Anti-PD-1 antibody)	Adult, Older adult	Phase1	154	/
	NCT05407441	Tazemetostat+Nivo/Ipi in INI1-Neg/SMARCA4-Def Tumors	Tazemetostat (EZH2 Inhibitor), Nivolumab (Anti-PD-1 antibody), Ipilimumab (Anti-CTLA-4 antibody)	Child, Adult	Phase1, Phase2	49	Combination with EZH2 Inhibitor, Tazemetostat.
	NCT02989636	Nivolumab With or Without Stereotactic Radiosurgery in Treating Patients with Recurrent, Advanced, or Metastatic Chordoma	Nivolumab (Anti-PD-1 antibody), Stereotactic Radiosurgery	Child, Adult, Older adult	Phase1	21	Combination with Stereotactic Radiosurgery
	NCT05286801	Tiragolumab and Atezolizumab for the Treatment of Relapsed or Refractory SMARCB1 or SMARCA4 Deficient Tumors	Tiragolumab (Anti-TIGIT antibody), Atezolizumab (Anti-PD-L1 antibody)	Child, Adult, Older adult	Phase1, Phase2	86	/
	NCT03190174	Nivolumab (Opdivo) Plus ABI-009 (Nab-rapamycin) for Advanced Sarcoma and Certain Cancers	Nab-Rapamycin (mTOR Inhibitor), Nivolumab (Anti-PD-1 antibody)	Child, Adult, Older adult	Phase1, Phase2	34	Combination with mTOR Inhibitor, ABI-009.
	NCT02834013	Nivolumab and Ipilimumab in Treating Patients With Rare Tumors	Ipilimumab (Anti-CTLA-4 antibody), Nivolumab (Anti-PD-1 antibody)	Adult, Older adult	Phase2	818	/
Vaccine	NCT03595228	BN Brachyury and Radiation in Chordoma	BN-Brachyury Radiation	Child, Adult, Older adult	Phase2	29	Combination with radiation.
	NCT02383498	QUILT-3.011 Phase 2 Yeast-Brachyury Vaccine Chordoma	GI-6301 Placebo, GI-6301 Vaccine (Yeast- Brachyury), Radiotherapy	Adult, Older adult	Phase2	55	Combination with radiation.
	NCT04246671	TAEK-VAC-HerBy Vaccine for Brachyury and HER2 Expressing Cancer	TAEK-VAC-HerBy (Expressing Brachyury and HER2)	Adult, Older adult	Phase1	55	/
Oncolytic Virus	NCT00931931	HSV1716 in Patients with Non-Central Nervous System (Non-CNS) Solid Tumors	HSV1716	Child, Adult	Phase1	18	/
	NCT04091295	BLESSED: Expanded Access for DeltaRex-G for Advanced Pancreatic Cancer and Sarcoma	DeltaRex-G	Child, Adult, Older adult	/		
	NCT03886311	Talimogene Laherparepvec, Nivolumab and Trabectedin for Sarcoma	Talimogene Laherparepvec (A novel Oncolytic Virus), Nivolumab (Anti-PD-1 antibody), Trabectedin (A chemotherapy drug)	Adult, Older adult	Phase2	40	Combination with Nivolumab or Trabectedin.
Others	NCT01924689	Safety Study of Intratumoral Injection of Clostridium Novyi-NT Spores to Treat Patients with Solid Tumors That Have Not Responded to Standard Therapies	Clostridium novyi-NT spores (A kind of designed bacterium)	Adult, Older adult	Phase1	24	/

### 3.1 Vaccine therapy

Chordoma cells specifically expressed Brachyury, which was one of the hallmark features of chordoma and constituted a target for chordoma vaccine therapy (48). In 2015, Christopher designed a yeast-derived Brachyury vaccine and conducted a Phase I clinical trial on chordoma. The trial enrolled 34 patients, designed a 3 + 3 dose escalation experiment with three dose gradients, observed the side-effects and Brachyury-specific T cell responses to access the safety and efficacy of the vaccine. The results showed that there were no autoimmune reactions or severe adverse events in all patients, and a transient Brachyury-specific T cell production was observed in most evaluable patients, indicating a good safety profile and potential efficacy (15). Based on this result, Peter et al. conducted a double-blind randomized placebo-controlled Phase II clinical trial of the vaccine on chordoma (NCT02383498). The trial enrolled 24 patients with locally advanced chordomas which were ineligible for surgical resection, and the patients were given a yeast-derived Brachyury vaccine in combination with standard radiotherapy to observe the prognosis. However, the results showed that although Brachyury-specific T cell responses were present in the treatment group, the median survival was unexpectedly shorter than that in the control group (20.6 months vs. 25.9 months), which leading to the termination of this trial (16).

Although this clinical trial failed, it did not dissuade researchers from designing new Brachyury vaccines. Peter et al. conducted another Phase I clinical trial of a recombinant carrier Brachyury vaccine (MVA-BN-Brachyury-TRICOM vaccine) and the results showed good safety and significant Brachyury-specific T cell responses for this carrier Brachyury vaccine (49). At present, the Phase II clinical study of this BN-Brachyury had also been completed (NCT03595228). It enrolled 29 patients with chordoma to evaluate whether BN-Brachyury vaccine combined with radiotherapy could improve the objective radiological response rate (ORR) of patients, and the relevant results had not yet been published. Expect for this study, another Phase I clinical trial of Brachyury vaccine was ongoing (NCT04246671). The experiment used a novel recombinant virus TAEK-VAC-HerBy which expressed both Brachyury and HER2 peptides to stimulate the immune system and activate the anti-tumor immunity. The trial was currently recruiting patients, and the results had not yet been observed.

In addition to Brachyury vaccine therapy, there had been studies using irradiated autologous tumor cells as vaccine to treat chordomas. In 2015, Denis et al. subcutaneously implanted irradiated autologous tumor cells in a 49-year-old male skull base chondroid chordoma patient (NCT02193503). The patient received a total of five injections over a nine-week period, after which time the patient experienced no systemic toxicity and a delayed-type hypersensitivity reaction to the patient’s autologous tumor cells. Two months after vaccination, the tumor showed continuous shrinkage, the mucosal invasive tumor completely disappeared and the skull base and facial tumors showed long-term reduction, showing the good effect of the autologous tumor vaccine (50).

The results mentioned above indicated that the vaccine therapy for chordoma might be effective. However, there were still very few studies at present, and the data obtained were very limited. More clinical trials were needed to screen safe and effective vaccines for chordoma.

### 3.2 ICI therapy

ICI therapy had shown good results in a variety of tumors. In chordoma, the published literature on ICI therapies were mostly sporadic case reports, and clinical cohort studies were rare. The first ICI study in chordoma was reported in 2017. Denis treated 2 chordoma patients with PD-1 monoclonal antibody, including one conventional chordoma with metastases and one dedifferentiated chordoma with local recurrence. Although IHC staining showed low expression of the PD-1/PD-L1 in both cases before the ICI treatment, both of them received clinical and imaging remission after ICI treatment, and one of them showed a significant increase in PD-L1 expression after (50). Andrew et al. reviewed the cases of 17 patients with recurrent chordomas who received ICI therapy from 2016 to 2020, most of whom received Pembrolizumab (n=9, 53%) treatment. The results showed that the safety of ICI therapy was acceptable, but 2 patients experiencing grade 3/4 immune-related toxicity. In terms of efficacy, most patients had clinical benefits (n=15, 88%), including complete remission (CR, n=1, 6%), partial remission (PR, n=3, 18%) and stable disease (SD, n=11, 65%). Among all responders (n=15), the median response duration was 12 months. The 1-year OS rate was 87%, the 1-year PFS rate was 56% and the median PFS was 14 months (51). Carine et al. reviewed ICI therapies for SMARCB1-deficient sarcomas, including 1 case of atypical chordoma whose tumor shrunk significantly after receiving PD-1 inhibitor treatment (52). The results were encouraging, indicating that ICI therapy had a high clinical benefit rate for patients with recurrent chordomas.

In contrast to the above encouraging results, a Phase II clinical study on PD-L1 monoclonal antibody combined with CTLA-4 monoclonal antibody for advanced or metastatic bone tumors showed that among the 5 chordoma patients included in the study, only 1 patient had obvious tumor shrinkage and achieved CR after receiving a full course of twelve cycles (53). Xiang et al. conducted a systematic review of 22 chordoma cases involving progression from 2015 to 2022 and found that combination therapy with PD-1/PD-L1 antibodies and CTLA-4 monoclonal antibody was no more effective than monotherapy with PD-1/PD-L1 antibodies, but it was more toxic (54). However, due to the small number of cases and inconsistent drug regimens, the reliability of this result needed to be further verified. At present, there were two Phase II clinical trials (NCT04416568, NCT02834013) of PD-1 inhibitors and CTLA-4 inhibitors in the treatment of chordoma ongoing, and the efficacy of them was yet to be reported.

Reviewing the existing clinical data of ICI treatment of chordoma, we thought that ICI therapy might be effective for chordoma, and the choice of treatment plan might have a great impact on the treatment effect. However, it was still difficult to draw conclusions about when chordomas should receive ICI therapy and what treatment plan was optimal because of the limited ICIs therapies cases. Multiple ICI combination regimens, including two-ICI or multi-ICI combination, ICI combined with radiotherapy, ICI combined with chemotherapy were currently in clinical trials, and the relevant results would further deepen our understanding of chordoma immunotherapy.

### 3.3 Other immunotherapies

Some other immunotherapy options were on the clinical trials, such as CAR-T therapy or oncolytic virus therapy but had not yet been reported in clinical research on chordoma.

For CAR-T therapy, Long et al. had designed a kind of B7-H3 targeted CAR-T and verified its efficacy *in vitro*, but no further clinical trials had been registered (45). And regarding the oncolytic virus, there were three oncolytic virus clinical trials (NCT00931931, NCT00931931, NCT03886311) were in progress and relevant experimental results had not yet been published.

In addition to the above-mentioned common immunotherapy regimens, there was another rare immunotherapy clinical trial ongoing, a Phase I clinical trial (NCT01924689) enrolled 1 case of chordoma patient and studied the safety of the intratumoral injection of *Clostridium novyi-NT spores* in solid tumors which were not sensitive to standard treatment. *Clostridium novyi-NT spores* were an attenuated strain of *C. novyi* that lacked the lethal alpha toxin. When administered intravenously or intratumorally, *C. novyi-NT spores* could colonize and replicate in hypoxic regions of the tumor, triggering strong, precise, and tumor-limited cell lysis and immune-inflammatory responses, resulting in tumor death (55, 56). In this Phase I clinical trial, there were 8 cases of grade 3/4 adverse reactions in all 24 enrolled patients and the chordoma was stable for efficacy assessment after treatment (57). Based on the safety and efficacy of this study, we believed that the safety of this immunotherapy might still need to be further improved, and there was still a long way to go.

Combining the immunotherapy studies results mentioned above, we could see that currently, the only immunotherapy with significant research outcomes for chordoma was vaccine therapy. This was mainly because the presence of the tumor-specific antigen, Brachyury, in chordoma, which provided a clear target for vaccine research. Therefore, it was theoretically feasible to develop a vaccine targeting Brachyury to treat chordoma. The focus of current research on Brachyury vaccine was how to stimulate T cells more effectively to achieve a tumor-killing effect.

In addition, ICI therapy, due to its numerous selectivity options, was part of the widely conducted clinical trials. However, research reports on its safety and drug combinations were controversy and lacking in large-scale studies. In future research, the safety issue must be addressed first in order to continue further studies. Overall, vaccine therapy and ICI therapy were the main directions for future immunotherapy of chordoma.

## 4 Conclusion

Through a review of studies on the immune microenvironment and immunotherapy for chordoma, we found that current studies were still relatively limited and narrowly scoped.

For the immune microenvironment of chordoma, CD4+T cells and macrophages were currently believed as the most abundant infiltration subsets of immune cells, a variety of cytokines and chemokines were high secretion and multiple immune checkpoints such as PD-1, PD-L1 and CTLA-4 were high expression there, which had important impact on tumor progression and patient prognosis. However, some existing contradictory research results needed to be further studied, interpreted, and analyzed, and the production mechanism of immune cell infiltration, cytokine secretion, immune checkpoint expression and its interaction mechanism with tumor cells was still blank and needed to be further demonstrated.

For the immunotherapy of chordoma, vaccine therapy and ICI therapy were currently being developed, but no breakthrough progress had been made. Other immunotherapy studies, such as oncolytic virus therapy or CAR-T therapy, were still mainly in the preclinical stage, with only a few clinical trials underway. Overall, there were few published clinical studies on immunotherapy of chordoma and there was a lack of effective pooled analysis. Although clinical trials were underway, there were still insufficient types (e.g., no CAR-T clinical trials) and a small number (only 16 clinical trials), which still needed to be further expanded.

In summary, immunotherapy for chordoma still had a long way to go, and it was necessary to carry out joint research from both basic and clinical aspects to accelerate the pace of immunotherapy research for chordoma.

## Author contributions

YC: Writing – review & editing, Writing – original draft. HZ: Writing – review & editing.
